# Review of “*Malaria parasites*: *comparative genomics*, *evolution and molecular biology*” by Jane M. Carlton, Susan L. Perkins and Kirk W. Deitsch

**DOI:** 10.1186/1756-3305-6-74

**Published:** 2013-03-18

**Authors:** Paul Horrocks

**Affiliations:** 1 Keele University Medical School, Staffordshire, ST5 5BG, UK

## Book details

Carlton JM, Perkins SL, Deitsch, KW: *Malaria Parasites*: *Comparative Genomics*, *Evolution and Molecular Biology*. Caister Academic Press; 2013. 280 pages. ISBN 190823007X

## Review

Within the *Plasmodium* post-genomic era there have been two notable edited collections of reviews on the genomes and biology of malarial parasites; the first was a predecessor to this text edited by Andy Waters and Chris Janse (in 2004), the second was edited by Irwin Sherman (in 2005). Both of these sit on a shelf in my office, right next to this welcome addition to the series. It is only fair at the outset that I declare a guilty pleasure that I suspect I share with many of my peers – I *love* edited collections of reviews prepared by my more erudite colleagues. I justify this academically by appreciating their efforts in preparing concise, well-written reviews that surmise and appraise the current “state of the art” in their respective fields. And this text meets this requirement admirably. I suspect, however, that I take a similar amount of pleasure from the heft of a new academic tome (814g, hardback in a lovely dark magenta, slightly redolent of fish?) that offers a simple (lazy) “one-stop” information resource for the next few years of lecture preparation and discussions with my students.

Following on from the publication of the first two *Plasmodium* genomes just over ten years ago, our community has witnessed a revolutionary shift in our understanding of the biology and pathology of this devastating disease through the application of post-genomic technologies. Moreover, parallel efforts applied in the study of other apicomplexan parasites have provided us with a new facet to explore – comparative genomics. This text takes on the challenge of tackling this vast array of additional genomic information, now available from different strains and/or species, and integrating them into a series of accessible chapters that relate the impact of comparative genomics to a diverse range of topics.

The text can be broadly broken down into three parts. Chapters 1, 2 and 4 cover topics from the taxonomy and phylogeny of microsporidia, the architecture of apicomplexan parasites and resources available for their study, and a more detailed description of the diversity within the *Plasmodium falciparum* and *P*. *vivax* genomes. Chapters 3, 5, 6 and 11 explore biological insights gleaned from the application of post-genomic technologies such as next generation sequencing, genetic crosses coupled with expression quantitative trait loci (eQTL) mapping and the application of new forward and reverse genetic tools. The remaining chapters explore topics such as epigenetic mechanisms governing gene expression, the molecular basis of erythrocyte invasion, protein export and trafficking within the infected host erythrocyte and parasite-vector interactions. Given the actual range of topics covered within these eleven chapters, this list doesn’t give justice to the efforts of the 34 contributors and I offer my apologies for this. As a reader, I appreciated the timely and critical appraisal of their respective fields and their significant efforts in providing a comprehensive list of primary resources – with just over 1600 citations in total.

## Conclusion

Like any edited collection of reviews in a fast-evolving field, this text will date relatively quickly, necessitating expensive new volumes to be regularly released. Should the editors choose to take up this challenge, I would presumptuously suggest two areas for improvement. First, some additional development around the timely themes of drug and vaccine development would be welcome. Whilst touched upon, I did feel their omission in this text. And second, given the cost of this text, it is a shame that no colour was used in any of the figures. Whilst some 90% are adequately presented in the grayscale used throughout, the remaining 10% were mournfully represented – particularly as I have seen these in their full colourful glory when originally published. Nevertheless, for PhD students and researchers working with malaria parasites, this text represents an essential and eminently accessible resource for their work.

## Competing interests

The author declares no competing interests.

